# Bone tissue morphology of rat offspring lactationally exposed to polychlorinated biphenyl 169 and 155

**DOI:** 10.1038/s41598-020-76057-7

**Published:** 2020-11-04

**Authors:** Jana Brankovič, Janja Jan, Gregor Fazarinc, Milka Vrecl

**Affiliations:** 1 Institute of Preclinical Sciences, Veterinary Faculty, University of Ljubljana, Gerbičeva 60, Ljubljana, Slovenia; 2 Department of Dental Diseases and Normal Dental Morphology, Faculty of Medicine, University of Ljubljana, Hrvatski Trg 6, Ljubljana, Slovenia

**Keywords:** Bone, Histology

## Abstract

Polychlorinated biphenyls (PCBs) are ubiquitous, persistent, organic pollutants also considered endocrine-disrupting chemicals. Our study examined the effects of lactational exposure to nondioxin-like PCB-155 and/or dioxin-like PCB-169 on longitudinal femur growth at the distal epiphyseal growth plate (EGP) in young rats at three different ages [postnatal days (PNDs) 9, 22, and 42]. After delivery, lactating rats were divided into four groups (PCB-169, PCB-155, PCB-155 + 169, and control) and administered PCBs intraperitoneally. The femurs of offspring were used to estimate growth rate (µm/day), and histomorphometric analysis on the distal femur included the thickness of the EGP and zones of proliferation and hypertrophy with calcification. Stereometry was used to determine trabecular bone volume density. In the PCB-169 and PCB-155 + 169 groups, PCB-169 affected longitudinal bone growth in the early postnatal period by interfering with chondrocytes in the EGP zone of proliferation and, to a lesser extent, the zone of hypertrophy. Morphometric alterations in EGP structure diminished until puberty. A slow growth rate persisted in the PCB-169 group until PND 42, while in the PCB-155 group, a fast growth rate between PNDs 9 to 22 was significantly reduced between PNDs 22 to 42. Sterometric assessment showed decreased trabecular bone volume in the PCB-155 + 169 group compared with that in the control on PND 9 and increased in the PCB-169 group compared with that in the PCB-155 group on PND 42. To summarize, studied PCB congeners exerted congener- and age-dependent effects on femur growth rate and its histomorphometric characteristics.

## Introduction

Polychlorinated biphenyls (PCBs) are a group of persistent organic pollutants included in the list of endocrine-disrupting chemicals^1^. In Europe, their production and usage were banned more than 40 years ago, which enabled a stabilization of or, in some areas, a reduction in their levels in the environment and human diet, including mother’s milk^2^. Still, their presence in the environment leads to their bioaccumulation in predator wild animals, as demonstrated in a marine mammal study in which the biopsied skin and blubber tissue from 87.5% of stranded free-ranging dolphins were shown to contain PCB concentrations above the toxicity threshold for physiological effects^3^. PCBs exhibit high air concentration in industrial areas, which contributes to the adverse effects on health, including air pollution-induced bone damage^4^. Up to 52.9% of humans are estimated to be exposed to PCB levels above the tolerable weekly intake^5^. Studies have associated elevated levels of highly chlorinated PCBs (e.g., PCB-206, -180, -170, and -209) with increased mortality risk, especially from cardiovascular diseases, as well as other adverse health conditions (e.g., diabetes, obesity, hyperlipidemia, and atherosclerosis)^6^.

Previous studies have reported non- or mono-*ortho* coplanar PCBs (e.g., PCB-126) binding affinity for the aryl hydrocarbon receptor (AhR)^7,8^, through which they exert dioxin-like activities, while di-*ortho* PCBs (e.g., PCB-138, PCB-153, and PCB-180) demonstrate competitive binding to estrogen and androgen receptors and, thus, interference with sexual hormone-regulated processes^9,10^. Structural similarities between 17β-estradiol (E_2_) and PCBs were proposed by a three-dimensional quantitative structure–activity relationship approach^11^ and because of evidence that biphenyls possessing both ortho and para-substituents are capable of competing with E_2_ for estrogen receptor (ER) binding^12^. A previous study showed that selected PCBs, including PCB-155 (2,2′,4,4′,6,6′-hexachlorobiphenyl), competitively bind to the mouse uterine ER, although the estimated affinity of PCB-155 is > 300-fold lower compared with that of E_2_^13^. In contrast, the same study showed that PCB-155 significantly decreases E_2_-induced luciferase activity in human mammary gland-derived epithelial MCF7 cells, suggesting anti-estrogenic activity^13^.

Concerning bone tissue, environmental exposures to PCBs and other organochlorines may have sex-specific effect on bone mineral density in a population aged over 60^14^. In experimental rats, exposure to dioxin-like PCB-126 alters (i) tibial geometry and bone formation rate^8^, (ii) bone tissue composition^15^ and (iii) bone strength^16^. In utero or lactational exposure to non-dioxin-like PCB-180 results in a dose- and sex-specific skeletal and dental effects^17^. Specifically, sex-dependent effects on tibial growth were evident on postnatal day (PND) 84 and not on PND 35. Males showed dose-dependent increases in tibial length, whereas such an effect was only observed in female rats at a dose of 10 mg/kg body weight^17^. Sheep fetuses also exhibited sex-specific effects on bone geometry and mineral content induced mainly by exposure to PCB-118 and, to a lesser extent, PCB-153^18^. Our previous studies on dioxin-like PCB-169 and non-dioxin-like PCB-155 reported reduced body weight gain and impaired craniofacial growth in lactationally exposed young rats on PNDs 9 and 22^19^, alterations in femur bone geometry on PND 22^20^, and biomechanics and mineral composition on PND 42^21^. Alterations in the femur on PNDs 22 and 42 were primarily induced by PCB-169, while the changes observed in the combined exposure group (PCB-155 + PCB-169) were similar but less pronounced, which suggests that a nonplanar PCB-155 congener might reduce the PCB-169-mediated toxic effects in the bone^20,21^.

Growth in long bones occurs at the epiphyseal growth plate (EGP) next to the metaphysis in a process called endochondral ossification. Chondrocyte proliferation, hypertrophy, and apoptosis in the EGP depend on several factors such as genetics, nutrition, environment, and hormones, which influence endochondral ossification in either a paracrine or endocrine manner^22^. Among the hormones, estrogens exert a biphasic effect on epiphyseal growth. Their maximal stimulatory effect is observed at low levels, while higher levels are associated with epiphyseal maturation and fusion of the epiphysis during puberty^23^. The growth process is sensitive to harmful effects, especially in puberty^24^, which occurs at around 4–6 weeks of age in rats. Therefore, the architecture and thickness of the EGP, including its distinct zones and trabecular bone (TB) at the diaphysis, could be affected by endocrine-disrupting chemicals such as PCBs. Decreased proliferation, altered maturation of hypertrophic chondrocytes of the EGP, and reduced osteoclast activity could impair longitudinal bone growth (LBG) in young rats^25^. A study reported a decrease in EGP thickness due to reductions in the zone of proliferation (PZ) and especially the zone of hypertrophy in female mice treated with E_2_ or ERα-selective agonist 1,3,5-tris(4-hydroxyphenyl)-4-propyl-1H-pyrazole, which resulted in shorter tibias^26^. Hypothyroidism in young rats was also reported to induce disorganization of chondrocyte columns in the proliferative zones and decrease the number of hypertrophic chondrocytes in the EGP, thus causing reduced LBG and delayed ossification^27^. In rats, some bones retain the capacity for longitudinal growth throughout most of their lives, which means that the epiphyses of these bones remain unfused for approximately 30 and 15 months of age in males and females, respectively. Nevertheless, the bone growth rate decreases after 10 months of age for proximal tibial epiphysis^28^; therefore, the effects on the cells and the function of EGP should be studied before that point, preferably around puberty.

We previously showed that offspring body mass, which is a general indicator of growth, was decreased compared with that of control rats in the PCB-169 and PCB-155 + 169 groups from PND 9 onward^20^ and was accompanied by an alteration in prepubertal and pubertal rat femur length and biomechanics on PNDs 22 and 42^20,21^. Those data were not segregated by sex as previous reports revealed that the difference in growth rate between male and female rats began during the peripubertal period. The gender-dependent difference in body weight, i.e., males weighing more than females, became evident only after PND 33, whereas general growth in rats’ distal EGP of the radius showed no sex-dependent differences between PND 20 to 40^29^. Accordingly, the sex-specific effect of PCBs/environmental organochlorines on bone tissue was mostly reported either in older population^14^ or in older animals^17^. The present study aimed to histomorphometrically examine the effects induced by nondioxin-like PCB-155 and dioxin-like PCB-169, individually and in combination, on the architecture of the EGP and TB at the metaphysis and to correlate them with estimated growth rates in the juvenile rat femur.

## Results

### Relative LBG and daily growth rates from PNDs 9 to 42

Femur lengths in the exposed groups relative to those in the control group on PND 9 and growth rates per day between PNDs 9 and 22 and between PNDs 22 and 42 are summarized in Table 1. On PND 9, the femur lengths were comparable between groups (*p* > 0.05). On PND 22, the relative femur lengths were significantly decreased in the PCB-169 and PCB-155 + 169 groups compared with those in both the control and PCB-155 groups (*p* < 0.001). On PND 42, the relative femur lengths were significantly decreased in the PCB-169 group compared with those in the control (*p* < 0.001) and PCB-155 (*p* = 0.009) groups. Additionally, relative femur lengths were decreased in the PCB-155 (*p* = 0.006) and 155 + 169 (*p* = 0.004) groups compared with those in the control group. The growth rate per day was faster between PNDs 9 and 22 than between PNDs 22 and 42. In the first period (PNDs 9–22), the growth rates in the PCB-169 and PCB-155 + 169 groups were significantly decreased compared with those in the control (*p* < 0.05) and PCB-155 groups (*p* < 0.001). In the second period (PNDs 22–42), the growth rate remained low in the PCB-169 group (*p* = 0.003), while in the PCB155 + 169 group, it reached a level comparable with the control group. Interestingly, the PCB-155 group displayed the largest daily increase in the first period but did not significantly differ from the control group (*p* = 0.096). However, the opposite was observed in the second period when the growth rate was significantly lower than that in the control group (*p* < 0.001).

**Table 1:** Femur length relative to the control group on PND 9 and longitudinal growth rate from PNDs 9 to 42 of Wistar rat offspring in the different exposure groups.

	PND	Control	PCB-169	PCB-155	PCB-155 + 169	*p-*value
(n)	9	(12)	(12)	(9)	(9)	
	22	(10)	(15)	(14)	(13)	
	42	(7)	(7)	(7)	(5)	
**Femur length relative to the control group on PND 9**						
	9	1.00 ± 0.03	0.98 ± 0.03	0.99 ± 0.02	0.96 ± 0.05	ns
	22	1.59 ± 0.05	1.52 ± 0.03^a,b^	1.61 ± 0.03	1.50 ± 0.08^a,b^	< 0.001
	42	2.26 ± 0.03	2.08 ± 0.10^a,b^	2.17 ± 0.07^a^	2.12 ± 0.08^a^	< 0.001
**Growth rate (µm/day)**						
	PND 9–22	519 ± 44	474 ± 23^a,b^	551 ± 23	473 ± 75^a,b^	< 0.001
	PND 22–42	385 ± 19	326 ± 56^a^	317 ± 37^a^	357 ± 45	< 0.001

Values are given as mean ± SD. The number of femurs used (*n*) is given in parentheses.

The statistical significance of differences between groups was analyzed by one-way ANOVA followed by Bonferroni’s post hoc test.

*PND* postnatal days, *ns* not significant (*p* > 0.05).

*p* ≤ 0.05 versus: ^a^Control group; ^b^PCB-155 group.

### Histological and histomorphometric assessment of the distal femur epiphysis

**Figure 1:**
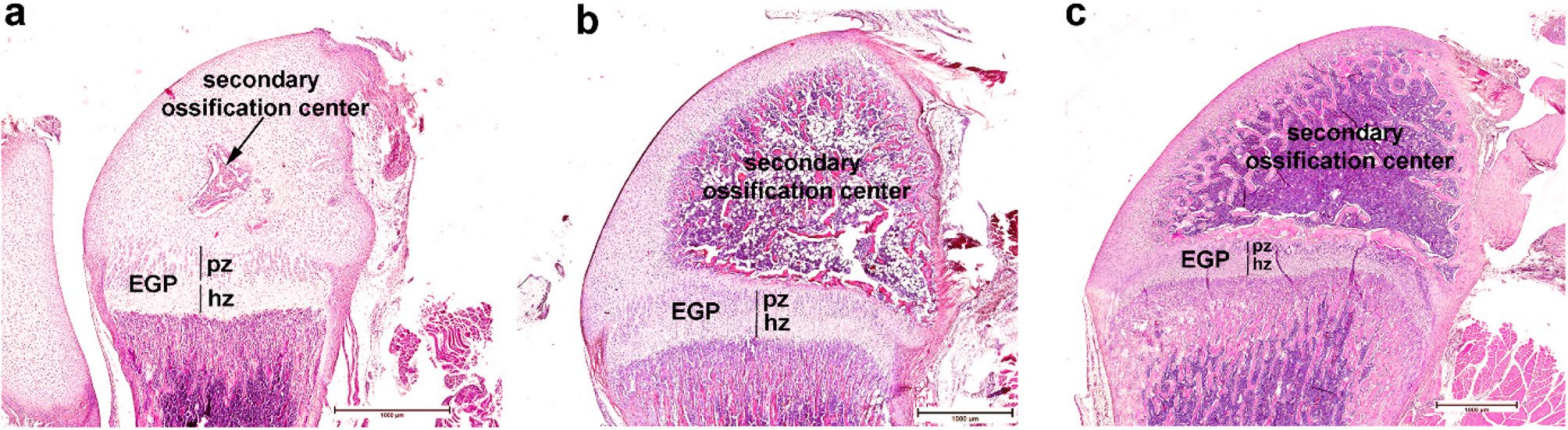
Endochondral ossification of the distal epiphysis of rat femur; (**a**) On PND 9, the secondary ossification center can be seen with blood vessel and connective tissue cell invasion and chondrocyte activation close to the ossification center. Surrounding chondrocytes are still in the resting phase; (**b**) on PND 22, the epiphysis is mostly ossified, and chondrocytes are present in the epiphyseal growth plate; (**c**) on PND 42, ossified epiphysis with EGP present. *EGP* epiphyseal growth plate, *pz* zone of proliferation, *hz* zone of hypertrophy and calcification, HE, scale bar 1000 µm.

On PND 9, the secondary center of ossification in the distal epiphysis was nascent. Hypertrophy of the central epiphyseal chondrocytes (secondary ossification center) could be seen, while the surrounding cartilage was still inactivated (zone of reserve cartilage) (Fig. 1a). On PND 22, the secondary center of ossification was fully developed, and the femur distal epiphysis was mostly ossified (Fig. 1b). On PND 42, the distal epiphysis was ossified, and chondrocytes were present in the EGP (Fig. 1c).

On PND 9, the thickness of the total distal EGP could not be determined, and therefore, only the absolute measures of the two EGP zones were analyzed (Fig. 1a). No alteration was observed in the cell architecture of the distal EGP, while the EGP-zone thickness differed between experimental groups (*p* ≤ 0.001). The PZ was decreased in the PCB-155 + 169 group compared with those in the control (*p* = 0.002) and PCB-155 (*p* = 0.005) groups. Compared with that in the control group, HZ was decreased in the PCB-169 (*p* < 0.001) and PCB-155 + 169 (*p* = 0.007) groups. Because of the decrease in HZ, the PZ/HZ ratio was significantly higher in the PCB-169 group than in the PCB-155 + 169 group (Table 2, *p* = 0.013).

**Table 2:** Thickness of femur distal EGP with its zones and their ratio of Wistar rat offspring on PND 9, 22 and 42 in the different exposure groups.

Parameter	PND	Control	PCB-169	PCB-155	PCB-155 + 169	p-value
(n)	9	(12)	(10)	(9)	(9)	
	22	(10)	(15)	(14)	(13)	
	42	(7)	(6)	(7)	(5)	
EGP thickness (μm)	22	596 (75)	552 (73)	610 (44)	599 (68)	ns
	42	413 (20)	433 (49)	331 (131)	398 (49)	ns
PZ (μm)	9	340 (41)	323 (48)^d^	328 (47)	290 (48)^a,b^	0.001
	22 ^¥^	246 (29)	210 (23)^a^	243 (26)	232 (19)	0.006
	42	153 (17)	169 (24)	137 (66)	145 (18)	ns
HZ (μm)	9	299 (28)	256 (28)^a^	266 (27)	250 (52)^a^	< 0.001
	22	287 (33)	273 (54)	284 (50)	283 (35)	ns
	42	197 (40)	209 (45)	174 (88)	205 (33)	ns
PZ/HZ	9	1.16 (0.21)	1.26 (0.26)^d^	1.25 (0.21)	1.07 (0.11)^c^	0.007
	22	0.87 (0.15)	0.75 (0.12)	0.83 (0.14)	0.80 (0.18)	ns
	42	0.79 (0.20)	0.85 (0.28)	0.79 (0.08)	0.76 (0.08)	ns

Values are given as median with IQR. The number of femurs used (*n*) is given in parentheses.

The statistical significance of differences between groups was analyzed by one-way ANOVA, followed by Bonferroni’s post hoc test or ^¥^by the Kruskal–Wallis test, followed by the Mann–Whitney post hoc test and Bonferroni correction.

*EGP* epiphyseal growth plate, *PZ* zone of proliferation, *HZ* zone of hypertrophy and calcification, *PND* postnatal days, *ns* not significant (*p* > 0.0167).

*p* ≤ 0.0167 versus: ^a^Control group; ^b^PCB-155 group; ^c^PCB-169 group; ^d^PCB-155 + 169 group.

On PND 22, cell architecture (Fig. 1b) and the thickness of the distal femur EGP did not differ between experimental groups (*p* = 0.059, Table 2). Compared with measurements obtained on PND 9, the PZ/HZ ratio changed (but not between groups, *p* = 0.071) as the HZ increased and prevailed over the PZ in all experimental groups. While the thickness of the HZ did not differ (*p* = 0.838), the thickness of the PZ was decreased in the PCB-169 group compared with that in the control group (*p* = 0.014) (Table 2). On PND 42, the EGP histomorphometric parameters did not differ between experimental groups (*p* > 0.05, Table 2).

### Stereometric assessment

**Figure 2:**
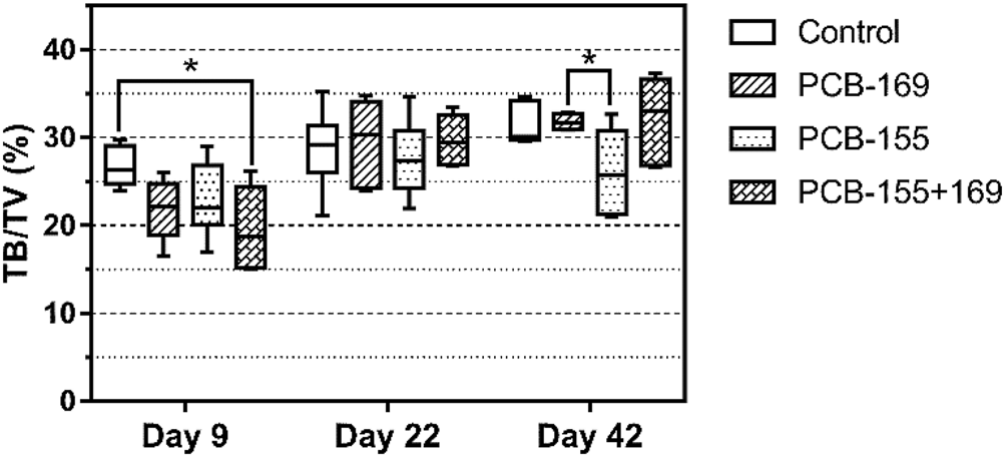
Trabecular bone volume of rat femur on PNDs 9, 22, and 42. Number of samples was *n* = 6, except for the PCB-155 + 169 group (*n* = 5). The statistical significance of differences between groups was analyzed by the Kruskal–Wallis test followed by the Wilcoxon rank-sum test. *TB* trabecular bone volume, *TV* total bone volume; *p < 0.05.

On PND 9, a decrease in the trabecular bone volume (TB/TV, %) was observed in the PCB-155 + 169 group compared with that in the control group (*p* = 0.014), while on PND 42, TB volume was higher in the PCB-169 group compared with that in the PCB-155 group (*p* = 0.041) (Fig. 2).

## Discussion

The present study examined the effects of lactational exposure to nonplanar PCB-155 and planar, dioxin-like PCB-169, individually and in combination, on longitudinal femur growth by histomorphometric and stereometric assessment of EGP architecture and TB at the metaphysis. Lactational exposure via mother’s milk was employed, as it is the main exposure route^5^ and offspring exposure to PCB congeners via mother’s milk has been previously confirmed; moreover, PCB levels in the offsprings’ serum were five-fold lower on PND 42 compared with those on PND 22^19,21^. The effects of PCB 169 and combined PCB-155 + 169 exposures on femur length relative to the control group on PND 9 were first observed on PND 22 and persisted until PND 42, while the inhibitory effect of nondioxin-like PCB-155 on relative femur length was first observed on PND 42. The latter was also accompanied by a significantly reduced daily growth rate in the period from PNDs 22 to 42. In contrast with the alterations in growth-related parameters (i.e., relative femur length and daily growth rate), which were first detected on PND 22, alterations at the microscopic level were already present on PND 9.

Our present data showed a rapid growth rate in rats at PNDs 9–22, which then decreased between PNDs 22–42. This growth rate dynamic followed the pattern previously described for rat proximal tibias and distal radii^29^ and is consistent with high serum estradiol levels in rats of both sexes between PNDs 9 and 21^30^. The effect on EGP zone thickness induced by PCB-169 alone (PNDs 9 and 22) or in combination with PCB-155 (PND 9) indicated impaired endochondral ossification in the distal femur epiphysis in the prepubertal period, which corroborated decreased growth rates in the first period (PNDs 9–22). On PND 9, decreased PZ and HZ in the PCB-155 + 169 group affected TB volume in the metaphysis without, up to that point, affecting the femur length. In the PCB-155 and -169 groups, reduced HZ by itself did not affect femur length or the volume density of newly formed TB underneath the growth plate. On PND 22, reduced relative femur length in the PCB-169 and PCB-155 + 169 groups^20^ displayed a correlation with the affected zones on PND 9 and with the decreased PZ in the PCB-169 group on PND 22. Similar to our observation, reduced LBG and delayed ossification^8^, together with alterations in EGP zones, were reported in laboratory animals previously^26,27^. Hypothyroidism also exerted an alteration in chondrocyte number and architecture in both the above-mentioned zones (i.e., disorganized columns of proliferated cells and reduced number of hypertrophic chondrocytes)^27^, while E_2_ and ERα agonist induced decreases in the HZ and, to a lesser extent, the PZ^26^. Yilmaz et al. also reported necrosis and increased production of hyaline cartilage in the TB of lumbar vertebrae as an indicator of elevated bone turnover, along with increased adipocyte content in marrow cavity in rats exposed to higher-chlorinated PCB mixture Aroclor 1254^31^.

Despite the lack of detectable histomorphometric alterations in the EGP on PNDs 22 and 42 in the PCB-155 group, the daily growth rate was significantly decreased in the second period after the onset of puberty. Similarly, we previously reported that an inhibitory effect of nondioxin-like PCB-155 on femur appositional growth was first observed on PND 42^21^. As the measured EGP parameters, but not relative femur length or estimated daily growth rate, became comparable with the control group level until puberty and the determined serum PCB levels were five-fold lower on PND 42 compared with those on PND 22, we postulate that the reversibility of these effects reflect the repair of growth function in EGP after gradual PCB reduction in the body^19^. However, at least in the PCB-169 group, a longer period would be required for alterations in femur growth-related parameters to be reversed. In support of the latter, a previous study reported bone alterations, such as shorter and narrower femurs or tibias on PND 35, which were then reversed by PND 77 and 350 in rat offspring exposed to Aroclor 1254 or an environmental contaminant mixture with 14 PCB congeners^32,33^. Similarly, a follow-up study of in utero and lactational 2,3,7,8-tetrachlorodibenzo-p-dioxin (TCDD) exposure demonstrated reversible effects on rat bones on PND 350 due to the clearance of TCDD from the body and repair of bone remodeling^34^.

In the PCB-155 + 169 group, the alterations observed at the microscopic level in the EGP on PND 9 were reflected in reduced LBG on PND 22^20^, and the effects observed in this group were ascribed to dioxin-like PCB-169^20,21^. Presumed stimulatory effect of PCB-155 on LBG manifested in significantly increased absolute femur length compared with that of the controls documented on PND 22, which was negated after adjusting for body mass^20^, was not accompanied by EGP histomorphometric alterations. Nevertheless, it was suggestive of the possible estrogenic potency of PCB-155 in the prepubertal period, while a significant inhibitory effect was observed afterwards. This result is quite intriguing and could be explained by low-affinity binding of PCB-155 to ER^13^, which has been reported to be expressed in rat tibial EGP from 1 to 40 weeks after birth^35^. Considering high E_2_ levels between PNDs 9–21^30^ and reported anti-estrogenic activity of PCB-155 in the presence of E_2_ by reporter gene assay in MCF7 cells^13^, reduced growth rate could be anticipated in the first period (PNDs 9–22), which was certainly not the case.

PCB-155 is one out of seven PCB congeners (PCB-77, -105, 114, -126, -156, and -169), the anti-estrogenic activity of which in vivo and in vitro evidence has been provided^36^. Certain congeners can also have tissue-specific effects in estrogen-dependent issues: coplanar PCB-126 was antiestrogenic in rat uterus and MCF7 cells and displayed weak estrogenic activity in bone tissue and the uterus from ovariectomized rats^37,38^. It could be assumed that certain PCBs may interfere with sexual hormone-regulated processes by binding (through AhR or constitutively active (androstane) receptor) to ER^9,10^ or via possible cross-talk between the ER-AhR signal transduction systems. This points to a very complex underlying mechanism that cannot be explained merely by a structure–function relationship and which warrants further studies.

Histological approaches have not been often utilized to assess the effect of PCB exposures on bone tissue^31,37^, although they could provide additional supporting evidence to better understand PCB-induced responses in bone tissue and correlate them with growth-related parameters. Evidence indicating associations between PCB exposure and some changes in bone parameters are also limited^39^. The shape of the distal femur EGP changes within the bone extremity; therefore, it is of utmost importance to use comparable bone samples (i.e., location) when performing histomorphometric analysis. There is an obvious time lapse between alterations observed at the microscopic level and growth-related disturbances, and reversibility of histomorphometric alterations and growth disturbances.

## Conclusions

Examination of the EGP in rat offspring on PNDs 9, 22, and 42, lactationally exposed to PCB-155 and -169 detected reversible alteration in the EGP PZ and, to a lesser extent, the HZ in groups exposed to PCB-169. Histomorphometric alterations disappeared until puberty, which suggests the repair of LBG function after gradual PCB reduction in the body. PCB-155 alone exerted effects in older animals after the onset of puberty and manisfested only in a significantly reduced growth rate. Lactational exposure to non-planar PCB-155 and planar PCB-169, individually or in combination, exerted congener- and age-dependent effects on femur growth rate and its histomorphometric characteristics.

## Materials and methods

### Experimental design

Nine-week old Wistar female rats (body mass 230–250 g) and one male rat were obtained from Lek d.d., Slovenia. Rats were raised conventionally in open polycarbonate cages, size 40 × 50 × 19 cm (Acrytech, Ljubljana, Slovenia), with wood chips for bedding (Lignocel, Rettenmaier & Söhne, Germany) under standard conditions (12-h light/dark cycle at 22 ± 2 °C and 40–60% humidity) and fed with a fixed-formula standard maintenance diet (Altromin 1324, Lage, Germany). Potable tap water was administered ad libitum. After mating, the females were housed individually until delivery when they were randomly divided into four experimental groups with their offspring. Rats were identified individually by non-invasive tail color marks.

**Figure 3:**
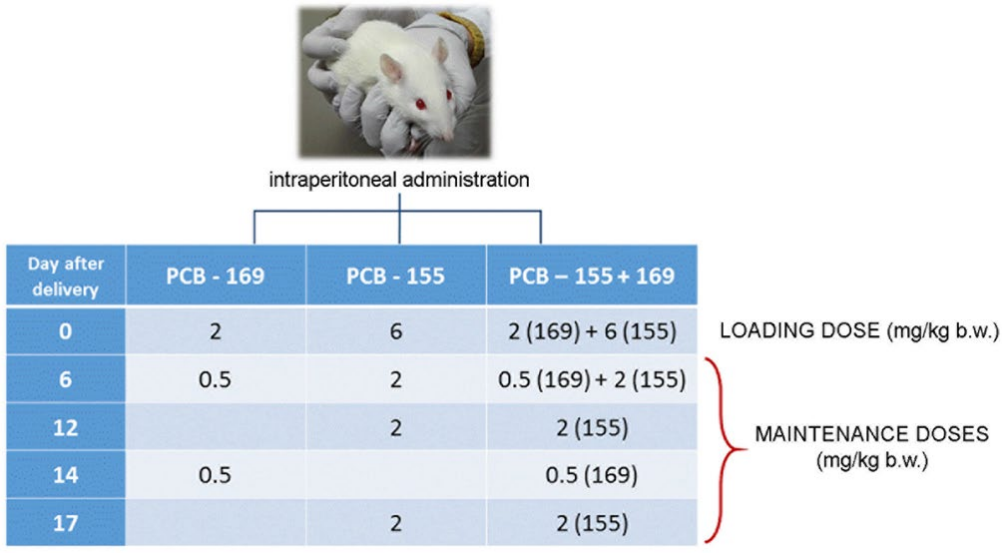
PCB administration regimen; day 0—the day of delivery.

PCB-155 and PCB-169 (Promochem, Wesel, Germany) were dissolved in olive oil and administered intraperitoneally to lactating rats. The PCB administration regimen, which included one loading and 2–4 maintenance doses, was previously described in detail^20,21^ and is schematically summarized in Fig. 3. The dose for PCB-169 was based on its TEF (0.03)^40^. The corresponding concentration of toxic equivalents (TEQs), an estimate of the total TCDD-like activity, was 90 μg TEQ/kg body mass. Comparable doses of PCB-169 were also used in some other studies^41,42^ and in a TCDD study with a comparable design^43^. To achieve comparable and constant levels of both PCBs throughout the exposure period, the administration regime with maintenance doses of PCB-155 and PCB-169 were determined according to the results of our previous study in which excretion patterns of (PCB -54, -80, -155, and -169) in ovine milk was determined^44^. Control animals were given an equal volume of olive oil. The loading dose was given to mothers on the day of delivery (day 0).

All animal experiments were assessed by an ethics committee, approved by the Administration of the Republic of Slovenia for Food Safety, Veterinary and Plant Protection, license number (3440-165/2006 and 34401-53/2011/2), and performed in accordance with the Slovenian Animal Protection Act (Official Gazette of the Republic of Slovenia, No. 43/2007) and the Council Directive 2010/63/EU.

Offspring were sacrificed in three postnatal periods: the suckling period on PND 9, the weaning period on PND 22, and the pubertal period on PND 42. The offspring that were euthanized on PND 9 consumed mother’s milk only. From PND 22, the offsprings’ diet was milk combined with solid food (Altromin 1324, Lage, Germany), as suckling began to decline around PND 20^45^, and from PND 28, offspring were separated from their mothers and consumed solid food only. The animals were anesthetized with ether and sacrificed by exsanguination.

### Femur bone size

Femurs were dissected and cleaned of soft tissue. On PND 9, right femurs were immediately fixed in formalin, while for the left bones, a Vernier sliding caliper was used to measure length (from the femur head to the distal point of the medial condyle) and diameter (at the narrowest part of the femur mid-diaphysis) to the nearest 0.05 mm. The relative LBG (%) was calculated between PNDs 9 and 22 and between PNDs 22 and 42 using the results from previous reports^20,21^. The growth rate (µm/day) was calculated for the periods between PNDs 9 and 22 and PNDs 22 and 42.

### Femur histomorphometry and stereometry

**Figure 4:**
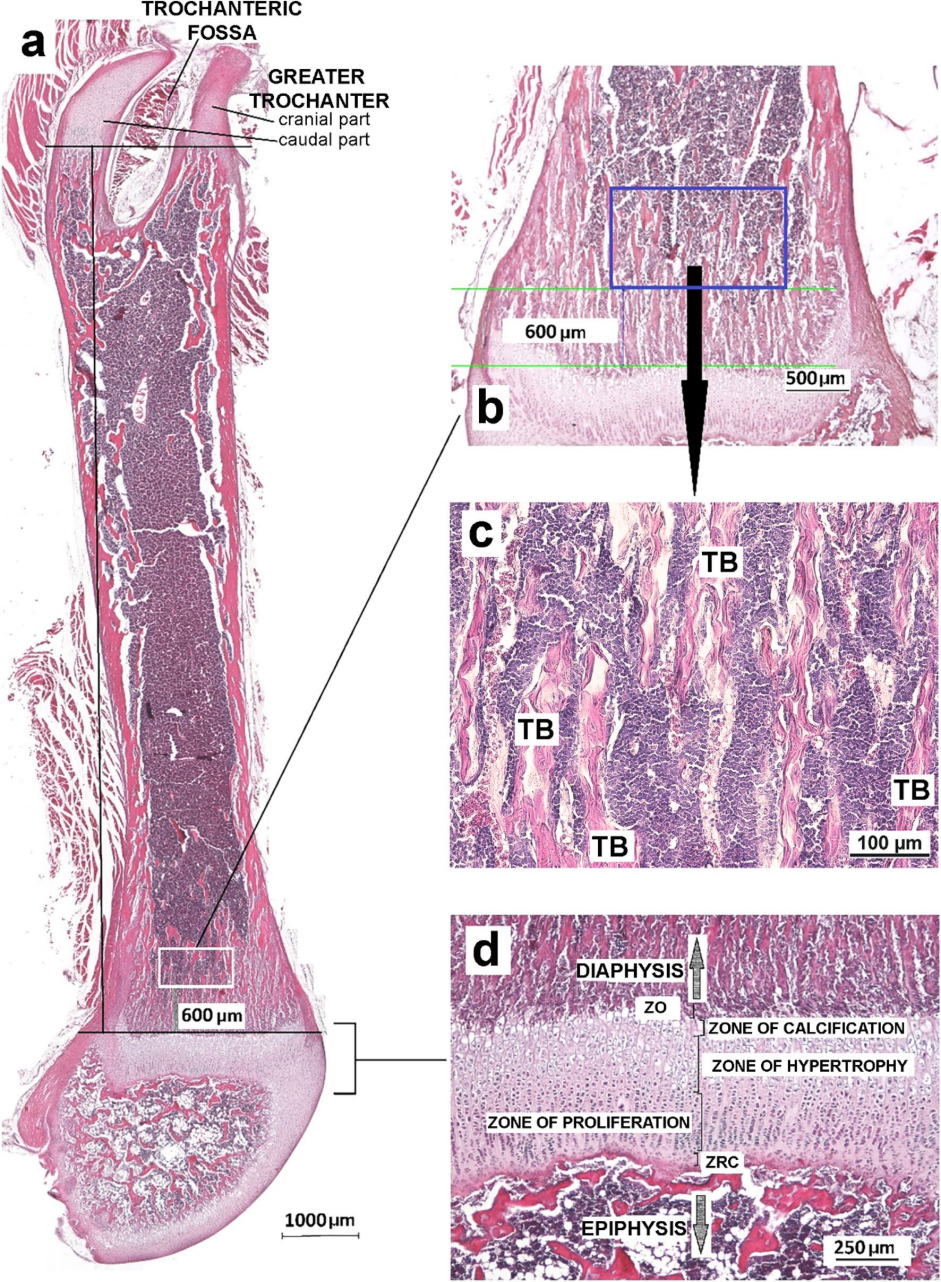
Representative photomicrographs of the femur from offspring on PND 22 used for histomorphometric analysis; (**a**) a micrograph overview of the longitudinal section of rat femur, hematoxylin and eosin (HE); (**b**) a higher magnification micrograph where the area marked with a white rectangle in panel a corresponds to the blue rectangle, representing the region of interest (1.4 × 0.8 mm) used for stereometry, 600 µm from the calcification zone of the distal epiphyseal growth plate (EGP), HE; (**c**) a higher magnification micrograph of trabecular bone (TB), HE; and (**d**) distal EGP with marked zones, *ZO* zone of ossification, *ZRC* zone of reserve cartilage, HE.

After fixation in 5% buffered formalin, right femurs were decalcified in 4.13% ethylenediaminetetraacetic acid solution (samples collected on PNDs 9 and 22) or Osteosoft solution (Merck, Darmstadt, Germany) (samples collected on PND 42) and embedded in paraffin. Five-micrometer tissue sections were cut (Leica SM2000 R Sliding Microtome, Nussloch, Germany) parallel to the longitudinal axis from the lateral to the medial side of the femur. Since the main nutrient foramen *(foramen nutricium)* could not be observed, we selected comparable longitudinal sections based on the position of the trochanteric fossa *(fossa trochanterica)* [i.e., at the location where the trochanteric fossa opens proximally and divides the greater trochanter (*trochanter major*) into its cranial and caudal parts] (Fig. 4a). Histomorphometric assessment was performed by a Nikon Eclipse Ni-UM Microscope equipped with a DS-Fi1 camera and Imaging Software NIS-Elements BR 4.60 (Nikon Instruments Europe B.V., Badhoevedorp, The Netherlands). Standardized nomenclature was used according to the American Society for Bone and Mineral Research^46^.

For histomorphometry of the distal femur epiphysis, sections were stained with hematoxylin and eosin (HE). Comparable longitudinal sections were used for histomorphometric measurements of the EGP [i.e., the thickness (in µm) of the distal EGP (on PNDs 22 and 42) its PZ, and zones of hypertrophy and calcification (HZ) (on PNDs 9, 22, and 42)] (Fig. 4d). The thickness of the distal EGP and HZ was measured at at least seven equally distanced positions and the thickness of the PZ at at least three (PND 22) or seven (PND 42) positions of the EGP. The thickness of the HZ and PZ relative to the thickness of the EGP (in %, on PNDs 22 and 42) and the ratio between the HZ and PZ were calculated.

TB volume in the femur (6 animals per experimental group in each age group) was determined as previously described^37^. Femur longitudinal sections were taken at two locations. The first one was at the level used for morphometric EGP analysis (Fig. 4a), and the second level was distanced 75 µm (PND 9) or 100 µm (PNDs 22 and 42) from the first one. The region of interest was distanced 200 µm (PND 9) or 600 µm (PNDs 22 and 42) from the zone of calcification (Fig. 4b,c). A 100-point grid was used to assess the volume density of TB in eight adjacent test fields per histological section. Volume density (%) was calculated by dividing the number of intersections of the above-mentioned structures by the number of all intersections, which corresponds to the total bone (TV) volume^37^.

### Statistical analysis

Significance was defined as *p* < 0.05. SPSS 20 for Windows (SPSS Inc., Chicago, IL, USA) was used for data analyses and GraphPad Prism 7.0 (GraphPad Software, San Diego, CA, USA) for data presentation. The differences between exposure groups were analyzed by one-way ANOVA followed by Bonferroni's post hoc test (normal distribution of the data) or, when necessary, by the Kruskal–Wallis test. In nonparametric tests, a Mann–Whitney test was used in post hoc analysis to determine the differences among the three PCB-exposed groups and the control, and a Bonferroni correction was calculated with *p* < 0.0167 as our level of significance.

## References

[1] WHO (2013). State of the Science of Endocrine Disrupting Chemicals—2012.

[2] Horvat M (2015). Monitoring Kemikalij in Njihovih Ostankov v Ljudeh za Leto 2011–2014: Zaključno Poročilo o Rezultatih Kemijskih Analiz.

[3] Genov T (2019). Linking organochlorine contaminants with demographic parameters in free-ranging common bottlenose dolphins from the northern Adriatic Sea. Sci. Total Environ..

[4] Prada D, Lopez G, Solleiro-Villavicencio H, Garcia-Cuellar C, Baccarelli AA (2020). Molecular and cellular mechanisms linking air pollution and bone damage. Environ. Res..

[5] Malisch R, Kotz A (2014). Dioxins and PCBs in feed and food—Review from European perspective. Sci. Total Environ..

[6] Lind PM, Salihovic S, Stubleski J, Karrman A, Lind L (2019). Association of exposure to persistent organic pollutants with mortality risk: An analysis of data from the prospective investigation of vasculature in uppsala seniors (PIVUS) study. JAMA Netw. Open.

[7] Van der Burght AS, Tysklind M, Andersson PL, Jean Horbach G, van den Berg M (2000). Structure dependent induction of CYP1A by polychlorinated biphenyls in hepatocytes of male castrated pigs. Chemosphere.

[8] Williams AE (2020). Skeletal toxicity of coplanar polychlorinated biphenyl congener 126 in the rat is aryl hydrocarbon receptor dependent. Toxicol. Sci..

[9] Bonefeld-Jorgensen EC, Andersen HR, Rasmussen TH, Vinggaard AM (2001). Effect of highly bioaccumulated polychlorinated biphenyl congeners on estrogen and androgen receptor activity. Toxicology.

[10] Pikkarainen A, Lehtonen M, Håkansson H, Auriola S, Viluksela M (2019). Gender- and dose-related metabolome alterations in rat offspring after in utero and lactational exposure to PCB 180. Toxicol. Appl. Pharmacol..

[11] Waller CL, Minor DL, McKinney JD (1995). Using three-dimensional quantitative structure-activity relationships to examine estrogen receptor binding affinities of polychlorinated hydroxybiphenyls. Environ. Health Perspect..

[12] Korach KS, Sarver P, Chae K, McLachlan JA, McKinney JD (1988). Estrogen receptor-binding activity of polychlorinated hydroxybiphenyls: Conformationally restricted structural probes. Mol. Pharmacol..

[13] Fielden MR, Chen I, Chittim B, Safe SH, Zacharewski TR (1997). Examination of the estrogenicity of 2,4,6,2',6'-pentachlorobiphenyl (PCB 104), its hydroxylated metabolite 2,4,6,2',6'-pentachloro-4-biphenylol (HO-PCB 104), and a further chlorinated derivative, 2,4,6,2',4',6'-hexachlorobiphenyl (PCB 155). Environ. Health Perspect..

[14] Hodgson S (2008). Bone mineral density changes in relation to environmental PCB exposure. Environ. Health Perspect..

[15] Ronis MJ (2020). Skeletal toxicity resulting from exposure of growing male rats to coplanar PCB 126 is associated with disruption of calcium homeostasis and the GH-IGF-1 axis and direct effects on bone formation. Arch. Toxicol..

[16] Lind PM (2000). Change of bone tissue composition and impaired bone strength in rats exposed to 3,3',4,4',5-pentachlorobiphenyl (PCB126). Toxicology.

[17] Romero AN (2017). Skeletal and dental effects on rats following in utero/lactational exposure to the non-dioxin-like polychlorinated biphenyl PCB 180. PLoS ONE.

[18] Gutleb AC (2010). Effects on bone tissue in ewes (*Ovies aries*) and their foetuses exposed to PCB 118 and PCB 153. Toxicol. Lett..

[19] Grošelj M (2015). Effects of lactational exposure to nonplanar PCB-155 and planar PCB-169 on body weight gain and craniofacial growth in rat offspring. Slv. Vet. Res..

[20] Brankovic J (2017). Alterations in geometry, biomechanics, and mineral composition of juvenile rat femur induced by nonplanar PCB-155 and/or planar PCB-169. Environ. Toxicol..

[21] Brankovic J (2019). Lactational exposure to dioxin-like polychlorinated biphenyl 169 and nondioxin-like polychlorinated biphenyl 155: Effects on rat femur growth, biomechanics and mineral composition. Ecotoxicol. Environ. Saf..

[22] Lui JC, Nilsson O, Baron J (2014). Recent research on the growth plate: Recent insights into the regulation of the growth plate. J. Mol. Endocrinol..

[23] Lui JC, Garrison P, Baron J (2015). Regulation of body growth. Curr. Opin. Pediatr..

[24] Shim KS (2015). Pubertal growth and epiphyseal fusion. Ann. Pediatric Endocrinol. Metab..

[25] Sanchez CP, He YZ (2009). Bone growth during rapamycin therapy in young rats. BMC Pediatrics.

[26] Iravani M, Lagerquist M, Ohlsson C, Savendahl L (2017). Regulation of bone growth via ligand-specific activation of estrogen receptor alpha. J. Endocrinol..

[27] Freitas FR (2005). The thyroid hormone receptor beta-specific agonist GC-1 selectively affects the bone development of hypothyroid rats. J. Bone Miner. Res..

[28] Jee WS, Yao W (2001). Overview: Animal models of osteopenia and osteoporosis. J. Musculoskelet. Neuronal Interact..

[29] Hansson LI, Menander-Sellman K, Stenstrom A, Thorngren KG (1972). Rate of normal longitudinal bone growth in the rat. Calcif. Tissue Res..

[30] Dohler KD, Wuttke W (1975). Changes with age in levels of serum gonadotropins, prolactin and gonadal steroids in prepubertal male and female rats. Endocrinology.

[31] Yilmaz B (2006). Modulatory effects of Aroclors 1221 and 1254 on bone turnover and vertebral histology in intact and ovariectomized rats. Toxicol. Lett..

[32] Elabbas LE (2011). In utero and lactational exposure to Aroclor 1254 affects bone geometry, mineral density and biomechanical properties of rat offspring. Toxicol. Lett..

[33] Elabbas LE (2011). Perinatal exposure to environmental contaminants detected in Canadian Arctic human populations changes bone geometry and biomechanical properties in rat offspring. J. Toxicol. Environ. Health A.

[34] Miettinen HM (2005). Effects of in utero and lactational TCDD exposure on bone development in differentially sensitive rat lines. Toxicol. Sci..

[35] van der Eerden BC, Gevers EF, Lowik CW, Karperien M, Wit JM (2002). Expression of estrogen receptor alpha and beta in the epiphyseal plate of the rat. Bone.

[36] Painter JT, Pinnella KD, Tessari JD, Cosma GN, Benjamin SA, Schantz S, Fischer L, Robertson LW, Hansen LG (2001). Hepatic enzyme induction and estrogen metabolism in PCB-exposed rats. PCBs: Recent Advances in Environmental Toxicology and Health Effects.

[37] Lind PM, Eriksen EF, Lind L, Orberg J, Sahlin L (2004). Estrogen supplementation modulates effects of the endocrine disrupting pollutant PCB126 in rat bone and uterus: Diverging effects in ovariectomized and intact animals. Toxicology.

[38] Cooke P, Sato T, Buchanan S, Robertson LW, Hansen LB (2001). Disruption of steroid hormone signaling by PCBs. PCBs: Recent Advances in Environmental Toxicology and Health Effects.

[39] Knutsen HK, EFSA (2018). Risk for animal and human health related to the presence of dioxins and dioxin-like PCBs in feed and food. EFSA J..

[40] Van den Berg M (2006). The 2005 World Health Organization reevaluation of human and Mammalian toxic equivalency factors for dioxins and dioxin-like compounds. Toxicol. Sci..

[41] Yamamoto M (2005). Effects of maternal exposure to 3,3',4,4',5-pentachlorobiphenyl (PCB126) or 3,3',4,4',5,5'-hexachlorobiphenyl (PCB169) on testicular steroidogenesis and spermatogenesis in male offspring rats. J. Androl..

[42] Xiao W (2011). Adverse effects of neonatal exposure to 3,3',4,4',5,5'-hexachlorobiphenyl on hormone levels and testicular function in male Sprague-Dawley rats. Environ. Toxicol..

[43] Lukinmaa PL (2001). Arrest of rat molar tooth development by lactational exposure to 2,3,7,8-tetrachlorodibenzo-p-dioxin. Toxicol. Appl. Pharmacol..

[44] Vrecl M, Ursic M, Pogacnik A, Zupancic-Kralj L, Jan J (2005). Excretion pattern of co-planar and nonplanar tetra- and hexa-chlorobiphenyls in ovine milk and faeces. Toxicol. Appl. Pharmacol..

[45] Baker HJ, Lindsey JR, Wesibroth SH, Baker HJ, Russell-Lindsey J, Wesibroth SH (1979). Biology and diseases. The Laboratory Rat.

[46] Parfitt AM (1987). Bone histomorphometry: Standardization of nomenclature, symbols, and units. Report of the ASBMR Histomorphometry Nomenclature Committee. J. Bone Miner. Res..

